# Circulating miRNA-29b and Sclerostin Levels Correlate with Coronary Artery Calcification and Cardiovascular Events in Maintenance Hemodialysis Patients

**DOI:** 10.1155/2021/9208634

**Published:** 2021-12-23

**Authors:** Jiqing He, Mingjiao Pan, Mingzhi Xu, Ruman Chen

**Affiliations:** Blood Purification Center, Hainan General Hospital, Hainan Affiliated Hospital of Hainan Medical University, Haikou, Hainan 570311, China

## Abstract

**Objective:**

Coronary artery calcification (CAC) is a common complication in end-stage renal disease (ESRD) patients undergoing maintenance hemodialysis (MHD), and the extent of CAC is a predominant predictor of cardiovascular outcomes in MHD patients. In this study, we sought to uncover the relationship between circulating miRNA-29b, sclerostin levels, CAC, and cardiovascular events (CVEs) in MHD patients.

**Methods:**

This study recruited patients receiving MHD for at least three months in the Hainan General Hospital between January 2016 and June 2019, and all patients were followed up 24 months for CVEs. The serum level of sclerostin was determined by enzyme-linked immunosorbent assay (ELISA) and miRNA-29b expression by real-time qPCR (RT-qPCR). All patients received cardiac CT scans to evaluate CAC, and CAC scores were expressed in Agatston units. The MHD patients with CACs <100 were arranged into the CAC (<100) group, those with 100–400 CACs into the CAC (100–400) group, and those with CACs >400 into the CAC (>400) group. Net reclassification index (NRI) and integrated discrimination index (IDI) were calculated to assess the predictive performance of serum sclerostin level for the occurrence of CVEs.

**Results:**

Compared with the CAC (<100) group, the CAC (>400) group had higher proportions of older patients, hypertension and diabetes mellitus patients, longer dialysis duration, higher mean arterial pressure (MAP), higher levels of high-sensitivity C-reactive protein (hs-CRP), alkaline phosphatase (ALP), and phosphate (*P* < 0.05). It was found that the CAC (100–400) and CAC (>400) groups exhibited higher serum levels of sclerostin but lower levels of miRNA-29b than the CAC (<100) group (*P* < 0.05) and the CAC (>400) group had a higher level of sclerostin and a lower level of miRNA-29b than the CAC (100–400) group (*P* < 0.05). The circulating level of miRNA-29b was negatively correlated with the serum level of sclerostin in MHD patients (*r* = −0.329, *P* < 0.01). The multivariate logistic regression analysis showed that hs-CRP, phosphate, sclerostin, and miRNA-29b were independent risk factors for CAC in MHD patients (*P* < 0.05, Table 2). ROC for prediction of CAC by sclerostin yielded 0.773 AUC with 95% CI 0.683–0.864 (*P* < 0.01). As depicted by Kaplan–Meier curves of CVE incidence in MHD patients according to median sclerostin (491.88 pg/mL) and median miRNA-29b (Ct = 25.15), we found that serum levels of sclerostin and miRNA-29b were correlated with the incidence of CVEs in MHD patients. When a new model was used to predict the incidence of CVEs, NRI 95% CI was 0.60 (0.16–1.03) (*P* < 0.05) and IDI 95% CI was 0.002 (−0.014 to 0.025) (*P* < 0.05), suggesting that sclerostin added into the old model could improve the prediction of the incidence of CVEs.

**Conclusions:**

These data suggest that circulating miRNA-29b and sclerostin levels are correlated with CAC and incidence of CVEs in MHD patients. Higher sclerostin and lower miRNA-29b may serve as independent risk factors for the incidence of CVEs in MHD patients.

## 1. Introduction

Chronic renal disease, as a clinical syndrome with evidence of injury of the renal structure and function, is characterized by irreversible and slow gradual evolution. Patients with this disease are asymptomatic in the early stage, and typical symptoms of renal insufficiency occur in the late stage. With the increase in hypertension, type 2 diabetes, and aging population, the number of chronic renal disease patients who rely on hemodialysis has rapidly increased worldwide [1, 2]. It was reported that, in the United States, over 600,000 patients with end-stage renal disease (ESRD) received maintenance hemodialysis (MHD) [3]. In fact, there were no clear guidelines that exist, which define ESRD. Clinically, ESRD is defined as renal failure [4]. Numerous studies have demonstrated that patients with ESRD are at a significantly higher risk of suffering from cardiovascular disease [5, 6], resulting in at least 10 times higher mortality than that of non-ESRD groups [2].

Coronary artery calcification (CAC) is very common in patients with chronic renal disease and more severe in patients with ESRD. CAC is an active process related to atherosclerosis, which is stimulated by the inflammatory pathway. Pathologically, CAC is characterized by the pathological deposition of mineral in the artery wall. It starts with microcalcifications with a size ranging from 0.5 to 15.0 *μ*m and develops into large calcium fragments, resulting in forming of flaky deposition of more than 3 mm [7]. The location, density, and confluence of calcifications may turn part of the arterial duct into a noncompliant structure [8]. In general, the lack of mineralization inhibitors such as osteopontin, fetuin, and *γ*-carboxyglutamic acid (Gla) protein and induction of osteogenesis are the main mechanisms of vascular calcification. The activity of osteoblast-type cells in bone formation leads to induction of osteogenesis [9]. The osteoblast-type cells, such as vascular smooth muscle cells, differentiate under the stimulation of oxidative stress and changes in bone morphogenetic protein or pyrophosphate levels. These cell differentiation has been proved to be involved in the occurrence of calcification [10, 11]. The intima and media of vascular smooth muscle cells produce matrix vesicles regulating mineralization [12].

It has been confirmed that sclerostin expression was associated with induced calcification in vascular smooth muscle cells [13, 14]. Sclerostin, as an osteocyte-specific protein, has become a marker of clinical and subclinical vascular diseases. Sclerostin is highly expressed in cardiovascular disease, diabetes, and chronic kidney disease. Aging populations are more likely to have a high level of sclerostin [15]. Sclerostin is a negative regulator of the Wnt signal related to cell development, differentiation, proliferation, polarity, and migration [16]. The Wnt signaling pathway helps to control the differentiation of mesenchymal stem cells, inhibit the differentiation of chondrocytes and adipocytes, and accelerate the differentiation of osteoblasts [17].

Sclerostin is the product of the SOST gene. It is a characteristic marker of bone cells and regulates bone formation and bone resorption [18]. MicroRNAs (miRNAs) are small noncoding single-stranded RNAs that post-transcriptionally regulate gene expression, which play an important biological function in cancer, cardiovascular disease, and diabetes [19] miRNA-targeted therapy that has made great progress in clinical conditions. It was found that miR-29b expression is downregulated in different cancers. It targets multiple genes in cell survival, angiogenesis, metastasis, fibrosis, and epigenetic modification pathways [20, 21]. A previous study indicated that the overexpression of miRNA-29b alleviates myocardial injury [22]. However, few studies have shown that the interaction between sclerostin and miRNA-29b affects vascular calcification and cardiovascular disease in patients receiving hemodialysis.

This prospective cohort study mainly explored the effect of the interaction between serum sclerostin level and miRNA-29b expression on aortic calcification in MHD patients and analyzed the correlation between aortic calcification and related outcome measures, such as mean arterial pressure (MAP), high-sensitivity C-reactive protein (hs-CRP), alkaline phosphatase (ALP), and phosphate, so as to provide a theoretical basis for the diagnosis and treatment of cardiovascular diseases in hemodialysis patients and improve patients' mortality.

## 2. Materials and Methods

### 2.1. Study Population

This prospective cohort study recruited patients receiving MHD for at least three months, aged 18 years or older and being able to provide consent in our hospital between January 2016 and June 2019. All patients were followed up for 24 months until June 2021. Patients who had undergone coronary artery bypass grafting or coronary artery stent implantation, with hepatitis B/C and other serious liver diseases, serious infections, malignant tumors, history of tumor bone metastasis, multiple myeloma or lower limb fracture in recent 3 months, active autoimmune diseases, active tuberculosis, peritoneal dialysis or renal transplantation switch to HD, glucocorticoid treatment in recent 3 months, and suffering from acute cardiovascular events a month before enrollment, were excluded from the study. All patients were given HD, 4 h for each time and three times a week, using the Fresenius 4008S machine (Fresenius, Bad Homburg, Germany), with a blood volume of 200–250 mL/min, a dialysate flow rate of 500 mL/min, and a single-pool urea clearance index (spKt/v) of 1.2 or more. Oral antihypertensive drugs were given as required for regulating calcium/phosphorus metabolism and correction of renal anemia. This study recruited 105 MHD patients consisting of 63 men and 42 women, with the age of (58.63 ± 10.50) years and dialysis duration of (40.57 ± 17.67) months. There were 50 cases of chronic glomerulonephritis, 30 cases of diabetic nephropathy, 9 cases of hypertensive renal damage, 5 cases of urinary acid nephropathy, 4 cases of chronic interstitial nephritis, 3 cases of polycystic kidney, 2 cases of obstructive nephropathy, 1 case of lupus nephritis, and 4 cases of unknown etiology. After the cardiac CT scan, the CACs of MHD patients were 210 (72, 504). According to CACs, the MHD patients were assigned into CAC (<100) group (*n* = 44), CAC (100–400) group (*n* = 27), and CAC (>400) group (*n* = 34). Written informed consent was obtained from each participant. The study protocol was approved by the Ethics Committee of Hainan General Hospital.

### 2.2. Study Design

Demographics and clinical characteristics of each participant were collected at the time of enrollment, including age, gender, primary diseases, body mass index (BMI), hypertension, diabetes mellitus, smoking history (>10 cigarettes per day lasting for 5 years or more), dialysis duration, Kt/V, and MAP. Blood samples were collected from MHD participants in the morning after overnight fast before midweek dialysis sessions for biochemical measurement. The serum hemoglobin (Hb) level was determined by using an automated hematology analyzer (Sysmex XN-9000, Japan), and the levels of creatinine, blood urea nitrogen (BUN), uric acid (UA), albumin, calcium, phosphate, ALP, total cholesterol (TC), triglycerides (TG), LDL cholesterol (LDL-C), HDL cholesterol (HDL-C), hs-CRP by using standard autoanalyzer techniques (Beckman AU5800), and the intact parathyroid hormone (iPTH) by using a UniCel DxI 800 Access Immunoassay System (Beckman Coulter, Inc., Fullerton, CA, USA) were also determined.

### 2.3. Serum miRNA-29b and Sclerostin Measurement

Total RNA was extracted from 200 *μ*l serum using the miRNeasy Serum/Plasma Kit (Qiagen, Germany) by strictly following the manufacturer's protocol. Synthesis of complementary DNA (cDNA) was performed using TaqMan MicroRNA Reverse Transcription Kit (Applied Biosystems, USA) under the following cycling conditions: 16°C for 30 min, 42°C for 30 min, and 85°C for 5 min. Synthesized cDNA was amplified on the ABI7500 Real-Time PCR Instrument (Applied Biosystems, USA) using Taqman PCR master mixture and target-specific TaqMan microRNA assay. The qPCR reaction was carried out according to the following cycling conditions: 95°C for 10 min followed by 40 cycles at 95°C for 15 s and 60°C for 1 min. The expression level of miRNA-29b was presented by relative fold change to U6 RNA based on the CT method. The RT primer, PCR primers, and TaqMan probe for miR-29b-3p were purchased from ABI. The serum level of sclerostin was determined by enzyme-linked immunosorbent assay (ELISA) using a commercially available kit (R&D Systems, USA) as per the manufacturer's protocol. The results were obtained from independent experiments in triplicate.

### 2.4. Cardiac CT and Coronary Artery Calcification Scores (CACs)

All patients received cardiac CT scans using the 64-slice spiral CT scanner (General Electric Healthcare, Milwaukee, WI, USA). The scan was electrocardiogram gated and began when the heart rates of patients were less than 70 times/min according to the protocol with a tube voltage of 120 kVp, tube current of 80 mA, 3.0 mm slice thickness, and displayed field of 1.5. Calcium deposits in the coronary arteries were independently identified by two radiologists with level 2 competence and over 9 years of experience in performing cardiac CT. The degree of CAC was reflected by CAC scores expressed in Agatston units (AUs) [23]. Total CAC scores were calculated after summing the CAC scores of the left main artery, the left anterior descending artery, the left circumflex artery, and the right coronary artery. The MHD patients with CACs <100 were arranged into the mild group, those with 100–400 CACs into the moderate group, and those with CACs >400 into the severe group.

### 2.5. Follow-Up and Cardiovascular Events (CVEs)

All patients were followed up every 6 months either by telephone or by outpatient visits for 24 months from the collection of blood samples. Follow-up end points were CVEs and death caused by cardiovascular disease. CVEs were defined as the incidence of congestive heart failure (HF) including HF with reduced ejection fraction (HFrEF), HF with midrange EF (HFmrEF) and HF with preserved ejection fraction (HFpEF) [24] (NYHA grade II or above according to clinical manifestations), arrhythmia [25] (according to clinical symptoms and ECG), angina pectoris [26] and myocardial infarction [27], sudden cardiac death [28], and cerebrovascular accidents, such as transient ischemic attack (TIA) [29], cerebral infarction [30], and intracerebral hemorrhage [31]. Each CVE was reviewed, adjudicated, and assigned an underlying cause by three physicians. The cardiovascular disease death was confirmed by the patient's medical history and death certifications.

### 2.6. Statistical Analysis

The sample size of the study was calculated by one-sample sensitivity and specificity power analysis (*β* = 0.90; *α* = 0.05) using PASS 11 software (“test for one sample sensitivity and specificity” of “diagnostic test (ROC)”). All result analyses were performed in the Statistical Package for the Social Sciences (SPSS) software program (version 21.0, USA). Before analysis, all data should be checked by the Kolmogorov–Smirnov test for distribution normality and then presented as either mean ± standard deviation for normally distributed continuous variables or median (interquartile range (IQR), 25%∼75%) for non-normally distributed continuous variables. The Mann–Whitney test was used for non-normally distributed continuous variables and Student's *t*-test for normally distributed continuous variables. The chi-square test was used to evaluate differences in prevalence. Spearman's correlation coefficient was used to test relationships between serum sclerostin and miRNA-29b levels. The Kaplan–Meier method was used to estimate the cumulative risk of CVEs in MHD patients. The area under the receiver operating characteristic (ROC) curve (AUC) was used as a performance measure of serum sclerostin, miRNA-29b levels, and CACs in predicting the occurrence of CVEs. Net reclassification index (NRI) and integrated discrimination index (IDI) were calculated to assess the predictive performance of serum sclerostin level for the occurrence of CVEs. NRI = [*P* (up/event) − *P* (down/event)] + [*P* (down/nonevent) − *P* (up/nonevent)]. Briefly, when calculating the NRI, a new model is considered superior if a higher risk is assigned to an individual with CVEs, and a lower risk to an individual without CVEs, compared with the old model [32]. The NRI value > 0.6 reflects strong model improvement, the value between 0.2 and 0.6 reflects moderate improvement, and the value less than 0.2 reflects weak improvement. The IDI represents the difference in discrimination slopes between the old and new models. The discrimination slope is the difference between the mean predicted risks in patients with CVEs versus those without CVEs [33]. Statistically significant differences were reflected by a value of *P* < 0.05.

## 3. Results

### 3.1. Clinical and Biochemical Characteristics of Included MHD Patients

As shown in Table 1, no significant difference was noted among the CAC (<100) group, CAC (100–400) group, and CAC (>400) group with regard to gender, smoking history, Kt/v, BMI, levels of Hb, TC, TG, HDL-C, LDL-C, UA, Alb, calcium, and iPTH (*P* > 0.05). Compared with the CAC (<100) group, the CAC (>400) group had higher proportions of older patients, hypertension and diabetes mellitus patients, longer dialysis duration, higher MAP, higher levels of hs-CRP, ALP, and phosphate (*P* < 0.05).

Serum levels of sclerostin and miRNA-29b were correlated with CAC in MHD patients.

**Figure 1:**
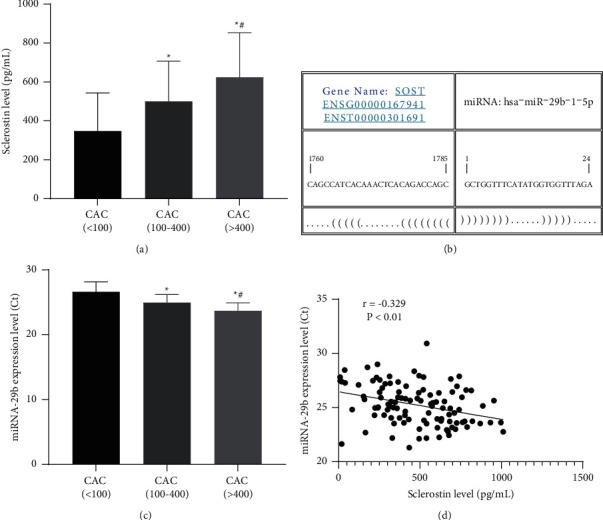
Serum levels of sclerostin and miRNA-29b were correlated with CAC in MHD patients. (a) The serum level of sclerostin among the CAC (<100) (*n* = 44), CAC (100–400) (*n* = 27), and CAC (>400) (*n* = 34) groups was determined by ELISA. (b) A miRNA-mRNA target prediction using the RNA22 database presented sclerostin as the target of miRNA-29b. (c) The serum expression level of miRNA-29b (Ct) among the CAC (<100) (*n* = 44), CAC (100–400) (*n* = 27), and CAC (>400) (*n* = 34) groups was determined by RT-qPCR. (d) A negative correlation between serum levels of sclerostin and miRNA-29b in MHD patients (*n* = 105).

Results of ELISA found that the CAC (100–400) and CAC (>400) groups exhibited higher serum levels of sclerostin than the CAC (<100) group (*P* < 0.05), and the serum level of sclerostin in the CAC (>400) group was higher than that in the CAC (100–400) group (*P* < 0.05, Figure 1(a)), suggesting that increased sclerostin levels were correlated with the incidence of CAC in MHD patients. We performed a miRNA-mRNA target prediction using the RNA22 database and reviewed sclerostin as the target of miRNA-29b (*P*=0.005, Figure 1(b)). Sclerostin functions as an inhibitor of the Wnt signaling, and miRNA-29b modulates Wnt signaling in human osteoblasts through a positive feedback loop [34]. MiRNA-29b was found to be reduced upon vascular smooth muscle calcification from previous evidence [35]. Subsequently, we performed RT-qPCR to determine the circulating level of miRNA-29b in MHD patients among the CAC (<100), CAC (100–400), and CAC (>400) groups. It was found that the CAC (100–400) and CAC (>400) groups exhibited lower serum expression levels of miRNA-29b than the CAC (<100) group (*P* < 0.05, Figure 1(c)), and the serum expression level of miRNA-29b in the CAC (>400) group was lower than that in the CAC (100–400) group (*P* < 0.05). To confirm the relationship between the circulating level of miRNA-29b and sclerostin in MHD patients, we performed Pearson's correlation analysis. Results revealed that the circulating level of miRNA-29b was negatively correlated with the serum level of sclerostin (*r* = −0.365, *P* < 0.001, Figure 1(d)).

Serum levels of sclerostin and miRNA-29b can be used as independent risk factors of CAC in MHD patients.

**Figure 2:**
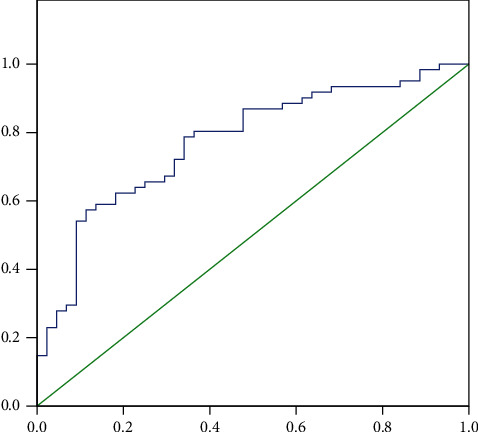
Receiver operating characteristic curves for prediction of CAC by serum levels of sclerostin.

With CAC = 100 as a cutoff for the presence of CAC, MHD patients were assigned into non-CAC and CAC groups. We performed a multivariate logistic regression analysis including age, hypertension, diabetes mellitus, dialysis duration, MAP, hs-CRP, ALP, phosphate, sclerostin, and miRNA-29b. Results showed that hs-CRP, phosphate, sclerostin, and miRNA-29b were independent risk factors for CAC in MHD patients (*P* < 0.05, Table 2). ROC for prediction of CAC by sclerostin yielded 0.773 AUC with 95% CI 0.683–0.864 (*P* < 0.01) as shown in Figure 2. The cutoff value of sclerostin was 491.88 pg/mL with a sensitivity of 83.23% and specificity of 63.12%.

Serum levels of sclerostin and miRNA-29b were correlated with CVEs in MHD patients.

**Figure 3:**
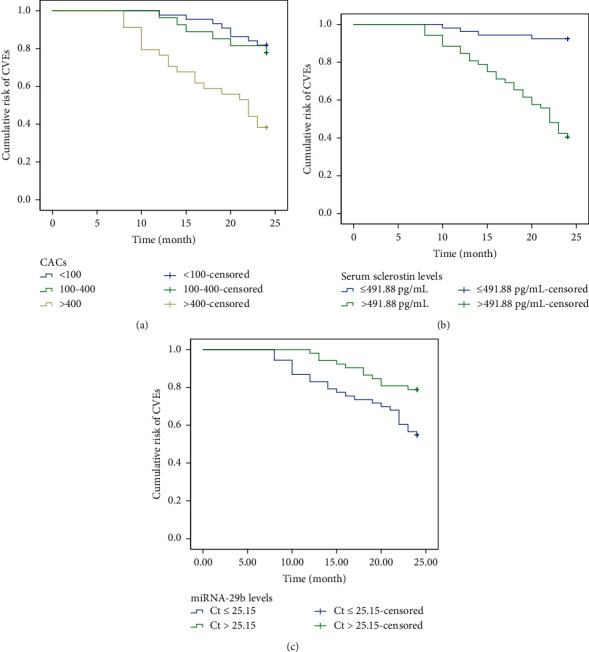
Serum levels of sclerostin and miRNA-29b were correlated with CVEs in MHD patients. (a) The Kaplan–Meier method with the log-rank test to estimate the cumulative risk of CVEs in MHD patients according to CACs. (b) The Kaplan–Meier method with the log-rank test to estimate the cumulative risk of CVEs in MHD patients according to serum levels of sclerostin (491.88 pg/mL as cutoff). (c) The Kaplan–Meier method with the log-rank test to estimate the cumulative risk of CVEs in MHD patients according to serum levels of miRNA-29b (Ct of 25.15 as cutoff).

Over a follow-up period of 24 months, among 105 MHD patients, there were 35 patients suffering from cardiovascular diseases with 53 CEVs, including 19 coronary artery diseases, 16 congestive HF (2 cases of HFrEF, 4 cases of HFmrEF, and 10 HFpEF), 10 cerebrovascular accidents (2 cases of TIA, 5 cases of cerebral infarction, and 3 cases of intracerebral hemorrhage), and 8 peripheral artery occlusion. It was found that there were 8 cases (18.18%) developing new-onset CVEs in the CAC (<100) group, 6 cases (22.22%) developing new-onset CVEs in the CAC (100–400) group, and 21 cases (61.76%) developing new-onset CVEs in the CAC (>400) group. A total of 6 MHD patients died of cardiovascular diseases, with 4 cases in the CAC (<100) group, 1 case in the CAC (100–400) group, and 1 case in the CAC (>400) group. Then, we used the Kaplan–Meier method with the log-rank test to estimate the cumulative risk of CVEs in MHD patients according to CACs. As shown in Figure 3(a), the cumulative risk of CVEs in MHD patients in the CAC (100–400) group was higher than that in the CAC (>400) and CAC (100–400) groups (*P* < 0.001). To determine the relationship between serum sclerostin levels and new-onset CVEs in MHD patients, we arranged 105 patients into sclerostin >491.88 pg/mL group and sclerostin ≤491.88 pg/mL group. The Kaplan–Meier method was used to estimate the cumulative risk of CVEs according to serum sclerostin levels. Results showed that the cumulative risk of CVEs in MHD patients in the sclerostin ≥491.88 pg/mL group was higher than that in the sclerostin <491.88 pg/mL group (*P* < 0.001, Figure 3(b)). Likewise, we arranged 105 patients into miRNA-29b (Ct) > 25.15 group and miRNA-29b (Ct) ≤ 25.15 group to determine the relationship between serum miRNA-29b levels and new-onset CVEs in MHD patients. As depicted by Kaplan–Meier curves and examined by log-rank test, the cumulative risk of CVEs in MHD patients in the miRNA-29b (Ct) ≤ 25.15 group was higher than that in the miRNA-29b (Ct) > 25.15 group (*P* < 0.001, Figure 3(c)). When a new model was used to predict the incidence of CVEs, NRI 95% CI was 0.60 (0.16–1.03) (*P* < 0.05) and IDI 95% CI was 0.002 (−0.014 to 0.025) (*P* < 0.05), suggesting that sclerostin added into the old model could improve the prediction of the incidence of CVEs.

## 4. Discussion

The incidence rate of chronic renal disease is increasing year by year. There are many risk factors, such as diabetes, hypertension, and IgA nephropathy, which are associated with the progression of chronic renal disease. These diseases can lead to renal function damage, eventually resulting in ESRD [36, 37]. ESRD patients mainly maintain their life through renal replacement therapy containing hemodialysis, peritoneal dialysis, and renal transplant [38]. Hemodialysis refers to a therapy that replaces the function of the kidney by diffusion, convection, adsorption, and ultrafiltration [39]. MHD patients are often complicated by vascular calcification. Vascular calcification is an important risk factor of cardiovascular disease and is associated with increased incidence rate and mortality of cardiovascular disease [40, 41]. The death risk of cardiovascular disease in MHD patients is at least 10-fold higher than that in the non-MHD population [2]. Therefore, the research on vascular calcification in patients with ESRD has attracted more and more attention.

Vascular calcification is a controllable and reversible pathophysiological process similar to the formation of bone and cartilage. Under the induced stimulation, vascular endothelial cells and smooth muscle cells can differentiate into chondroid cells, accompanied by calcium and phosphorus deposition in the vascular wall [42]. At present, some proteins such as vitamin K-dependent calcification inhibitory proteins [43], bone morphogenetic proteins [44], and sclerostin proteins [45] have been found to be involved in the regulation of vascular calcification.

Sclerostin is a secretory glycoprotein (22-kDa) encoded by the SOST gene and secreted by bone cells. It is the main inhibitor of the Wnt/beta-catenin signaling pathway and plays a key role in bone homeostasis [46] and vascular calcification [15]. Studies have shown that the expression of serum sclerostin in patients with chronic renal disease was higher than that in healthy population [47, 48]. This study divided the patients receiving MHD into three groups according to the degree of arterial calcification. The ELISA results in this study indicated that the patients with CAC (>400) showed significantly higher serum level of sclerostin than those in the patients with CAC (100–400) and CAC (<100). The patients with CAC (<100) indicated the lowest serum levels of sclerostin among the three groups. The findings were similar to the previous study suggesting that the increased expression of sclerostin was positively associated with higher odds of severe abdominal aortic calcification in MHD patients [49], and the research reported by Qureshi et al. revealed that the ESRD patients with a higher score of epigastric and coronary artery calcification showed much higher serum sclerostin levels [50]. At present, it is considered that the increase in serum sclerostin protein is due to the decrease in renal excretion caused by renal dysfunction. Pelletier et al. pointed out that the patients with stage III chronic renal disease revealed higher serum levels of sclerostin and that the increase in sclerostin was negatively correlated with glomerular filtration rate [51]. It has been suggested that the increase in sclerostin protein in chronic renal disease patients might be related to the increase in osteocyte production [52, 53]. Studies have confirmed that sclerostin is upregulated in the process of calcification of vascular smooth muscle cells in vitro [14]. The Wnt signaling pathway regulates the process of cell proliferation, cell differentiation, and tissue patterning. The activation of the Wnt signaling pathway contributes to the treatment of cardiovascular diseases [54]. Sclerostin, as an inhibitor, participates in the progression of vascular calcification by regulating the Wnt signaling pathway [54]. This study predicted that sclerostin was targeted by miRNA-29b using the RNA22 database and performed RT-qPCR to determine the circulating level of miRNA-29b among the three groups. It was observed that the patients with CAC (>400) manifested the lowest expression of miRNA-29b than the patients with CAC (100–400) and CAC (<100). The expression of miRNA-29b was the highest in patients with CAC (<100). In addition, Pearson's correlation analysis in the study confirmed that the expression of miRNA-29b was negatively correlated with sclerostin level. Sur et al. demonstrated that overexpression of miR-29b contributed to the inhibition of tumor growth of prostate cancer xenograft in nude mice [21]. The study presented by Pastuszak-Lewandoska et al. indicated that low expression of miRNA-29b was found in patients with nonsmall cell lung cancer [55]. All these results demonstrated that low expression of miRNA-29b is adverse to the prognosis of the disease. In order to analyze the risk factors related to the occurrence of CAC, this study assigned the MHD patients into non-CAC and CAC groups. The multivariate logistic regression analysis manifested that hs-CRP, phosphate, sclerostin, and miRNA-29b were the independent risk factors for the presence of CAC. A previous study revealed that the incidence of artery atherosclerosis and all-cause mortality in MHD patients was correlated with increased sclerostin [56].

Vascular calcification is a common pathological manifestation of atherosclerosis, hypertension, diabetes mellitus, vascular injury, chronic kidney disease, and aging [57]. It is one of the important factors of the high incidence rate and mortality of cardiovascular and cerebrovascular diseases [58]. The 24-month follow-up data of this study indicated that fewer patients had CVEs, including coronary artery diseases, congestive cardiac failure, cerebrovascular accidents, and peripheral artery occlusion, in the CAC (<100) group compared to the remaining two groups. The proportion of CVEs was the highest in the CAC (>400) group. Besides, it was found that more patients died in the CAC (<100) group. The Kaplan–Meier method with log-rank test showed the highest cumulative risk of CVEs was found in the CAC (100–400) group, and the cumulative risk of CVEs was higher in the patients with higher sclerostin and lower miRNA-29b. However, Drechsler et al. indicated that a high level of serum sclerostin decreased short-term (18-month) cardiovascular mortality in patients undergoing hemodialysis and peritoneal dialysis, but there was no significant association between serum sclerostin and long-term (4-year) cardiovascular mortality [59]. Desjardins et al. revealed that elevated serum sclerostin appears to be associated with cardiovascular mortality in the absence of adjustment for propensity scores including age, phosphate, interleukin-6, renal disease stage, and p-cresyl sulfate [60]. All these results implied that vascular calcification induced the occurrence of cardiovascular diseases but no strong positive correlation between vascular calcification and cardiovascular mortality. Various factors affected mortality.

In conclusion, this study showed that there is a positive correlation between serum sclerostin and aortic calcification in MHD patients, and this correlation is targeted by miRNA-29b through the Wnt signaling pathway. Sclerostin is an independent risk factor for aortic calcification, and the determination of serum sclerostin in hemodialysis patients is helpful to prevent cardiovascular disease. In the future, according to the correlation between sclerostin and miRNA-29b, an in vitro model of vascular endothelial cell calcification can be established to functionally verify that miRNA-29b negatively regulates sclerostin and affects vascular calcification.

## Figures and Tables

**Table 1:** Clinical and biochemical characteristics of MHD patients among the CAC (<100), CAC (100–400), and CAC (>400) groups.

Item	CAC (<100) *n* = 44	CAC (100–400) *n* = 27	CAC (>400) *n* = 34
Age (year)	56.27 ± 11.48	57.89 ± 9.53	62.26 ± 9.10^*∗*^
Gender/male (%)	29 (65.91%)	15 (55.56%)	19 (55.88%)
Smoking history (%)	18 (40.91%)	11 (40.74%)	14 (41.18%)
Hypertension (%)	32 (72.73%)	24 (88.89%)	31 (91.18%)
Diabetes mellitus (%)	9 (20.45%)	8 (29.63%)	15 (44.12%)^*∗*^
Dialysis duration (month)	31.55 ± 15.34	41.05 ± 16.18^*∗*^	51.88 ± 15.18^*∗*^^#^
Kt/v	1.36 ± 0.21	1.34 ± 0.25	1.31 ± 0.24
BMI (kg/m^2^)	22.32 ± 2.89	22.44 ± 2.45	23.34 ± 2.66
MAP (mmHg)	107.52 ± 11.65	111.91 ± 13.13	116.21 ± 13.30^*∗*^
Hb (g/L)	120.76 ± 12.12	118.87 ± 11.09	119.84 ± 13.28
TC (mmol/L)	3.84 ± 1.07	3.87 ± 0.98	3.79 ± 0.99
TG (mmol/L)	2.11 ± 0.94	2.23 ± 0.68	2.29 ± 1.05
HDL-C (mmol/L)	1.04 ± 0.31	0.94 ± 0.36	0.92 ± 0.22
LDL-C (mmol/L)	2.09 ± 0.91	2.12 ± 0.79	2.06 ± 0.90
UA (umol/L)	388.95 ± 124.44	430.20 ± 89.75	428.37 ± 107.84
Alb (g/L)	41.23 ± 3.82	40.72 ± 3.42	42.46 ± 7.18
hs-CRP (mg/L)	5.88 ± 1.99	8.22 ± 1.79^*∗*^	9.79 ± 2.69^*∗*^^#^
ALP (U/L)	77.17 ± 25.17	82.66 ± 27.12	91.12 ± 29.67^*∗*^
Calcium (mmol/L)	2.43 ± 0.23	2.48 ± 0.32	2.53 ± 0.32
Phosphate (mmol/L)	1.75 ± 0.54	2.05 ± 0.63	2.26 ± 0.49^*∗*^
iPTH (pg/ml)	187.48 (79.61, 346.33)	223.77 (52.06, 364.05)	331.43 (138.54, 609.24)

^∗^indicates that the data are statistically significant compared with CAC (<100) group. # reveals that the data are statistically significant compared with CAC (100–400) group.

**Table 2:** Multivariate logistic regression model of risk factors for CAC in MHD patients.

Variables	*B*	SE	Wals	OR (95% CI)	*P*
Age	−0.07	0.053	1.782	0.932 (0.841–1.033)	0.182
Dialysis duration	0.037	0.026	1.939	1.037 (0.985–1.092)	0.164
Sclerostin	0.004	0.002	3.043	1.004 (1.000–1.008)	0.031
miRNA-29b	−0.875	0.312	7.844	0.417 (0.226–0.769)	0.005
Diabetes mellitus	0.73	0.979	0.556	2.075 (0.305–14.131)	0.456
Hypertension	2.09	1.468	2.028	8.086 (0.456–143.514)	0.154
MAP	0.02	0.045	0.196	1.02 (0.934–1.115)	0.658
hs-CRP	0.785	0.251	9.809	2.193 (1.342–3.584)	0.002
ALP	−0.004	0.014	0.075	0.996 (0.969–1.024)	0.785
Phosphate	2.109	0.827	6.504	8.24 (1.629–41.672)	0.011

## Data Availability

The data used to support the findings of this study are included within the article.
